# New appreciation for an old pathway: the Lands Cycle moves into new arenas in health and disease

**DOI:** 10.1042/BST20210579

**Published:** 2022-02-28

**Authors:** Valerie B. O'Donnell

**Affiliations:** Systems Immunity Research Institute, School of Medicine, Cardiff University, Cardiff CF14 4SN, U.K.

**Keywords:** lipids, membranes, phospholipids

## Abstract

The Lands Pathway is a fundamental biochemical process named for its discovery by William EM Lands and revealed in a series of seminal papers published in the Journal of Biological Chemistry between 1958–65. It describes the selective placement in phospholipids of acyl chains, by phospholipid acyltransferases. This pathway has formed a core component of our knowledge of phospholipid and also diglyceride metabolism in mammalian tissues for over 60 years now. Our understanding of how the Lands pathways are enzymatically mediated via large families of related gene products that display both substrate and tissue specificity has grown exponentially since. Recent studies building on this are starting to reveal key roles for the Lands pathway in specific scenarios, in particular inflammation, immunity and inflammation. This review will cover the Lands cycle from historical perspectives first, then present new information on how this important cycle forms a central regulatory node connecting fatty acyl and phospholipid metabolism and how its altered regulation may present new opportunities for therapeutic intervention in human disease.

## Discovery of Lands pathway by Bill Lands and colleagues

William (Bill) EM Lands was a Biological Chemist born in 1930 in Chillilcothe, Missouri. Following a BS in Chemistry in Michigan, a PhD in Biological Chemistry from Illinois, and one year as Post Doc Fellow in California Institute of Technology, he spent 25 years on faculty at University of Michigan, starting around 1956. It was during this time that his seminal work on lipid metabolism included his discovery of the Lands Pathway, ultimately named after his own work. This pathway describes how selective fatty acyl (FA) placement is accomplished by lipid acyltransferases and phospholipases during recycling of glycerophospholipids (GPL), or more simply phospholipids (PL). It was uncovered in a series of seminal articles, reproduced in part during the centenary of JBC in 2005 [1–6]. At the time of Lands’ first publication on this topic, Kennedy and co-workers had proposed that lecithins (a generic term for PL) and triacylglycerides (TAG) were made from common diacylglyceride (DAG) precursors, based on experiments using isolated mitochondria [7, 8]. Lands reasoned that if this were always true, that radioisotope labelling using ^14^C-acetate and ^14^C-glycerol should produce a predicable ratio of incorporation in both TAGs and PLs. However, this turned out not to be the case, with a far higher level of incorporation of ^14^C in the FA compartment of the PL pool than expected. This led him to propose that the formation of lysoPL from PL was not simply a degradative end reaction for PL metabolism, but that a cycle existed where by exchange of FA can take place, via a lysoPL intermediate [1]. Thus, the diglyceride unit of PLs was proposed to be metabolically different from TAGs. Lands went on to further characterise this phenomenon by showing that enzymatic acylation of lysoPL (first generated through the action of phospholipases) occurred in rat liver microsomes [2]. He characterised the positional specificity of this acylation process [4], and followed this by showing that diacylPE could be generated from lysoPE, having previously shown this held true for PC biosynthesis [5]. Next, he showed that rat liver enzymes readily acylate glycero-3-phosphate in a way that doesn't discriminate acyl positioning, unlike acylation of monoacylglycerides [9], that acyltransferase activities were tissue selective, and proposed this was due to the different acyl coenzymeA : phospholipid acyltransferase activities observed [3]. In some of these studies, Lands was also characterising the different acyl transferases in his laboratory applying spectrophotometric assays to protein fractions purified from liver microsomes [4, 5]. He differentiated the enzymes that acted on 1-acyl versus 2-acylphosphoglycerides by their sensitivity to inhibition by long chain thiol esters.

These seminal studies set the groundwork for our understanding of PL metabolism, and were then followed by several decades research charactering the large families of acyl transferases that mediate these reactions using FA-CoA ester and lysoPL substrates. Notably, the Lands cycle doesn't exist in isolation, but alongside other biosynthetic pathways such as Kennedy Pathway or CDP-choline pathway, identified in 1956 by Eugene Kennedy as the primary pathway for PC biosynthesis [8]. For an excellent summary of PL biosynthesis and remodelling pathways including Lands and Kennedy Pathways, see The LipidWeb, by Bill Christie [10]. Overall, the importance of the Lands Pathway lies in the fact that this process is responsible for the extensive remodelling that determines the FA composition and positional specificity in cellular and tissue PL pools. Ultimately this complexity is defined and maintained by the tissue expression pattern of proteins that show strong specificity for particular FA and PL headgroups as outlined below.

## The enzymatic transformations that support the Lands pathway

**Figure 1.**
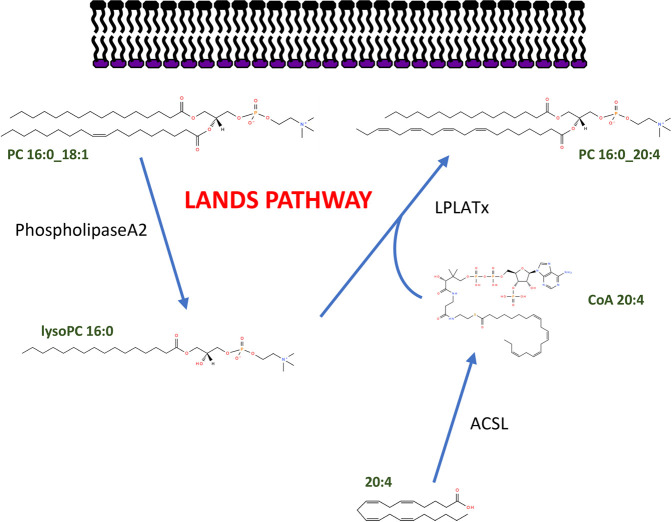
The Lands Pathway showing remodelling of PC fatty acyl composition. The cycle showing hydrolysis and re-esterification is shown as a summary.

While the reaction of the Lands Pathway may appear relatively straightforward, comprising the esterification of two substrates to form a single product, in reality this is far more complex with the different gene products showing a high level of specificity for particular FAs and PLs, leading to a hugely complex pattern of tissue specific PL composition. Furthermore, there are several mechanisms by which this can be accomplished, as outlined below. A summary figure of the Pathway is shown in Figure 1.

**Figure 2.**
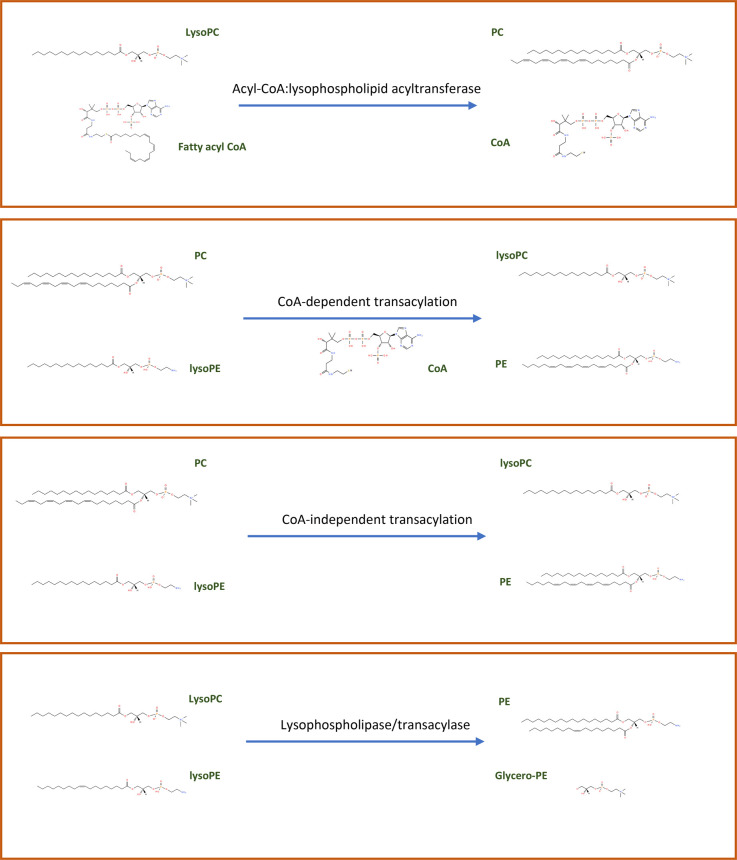
The four enzymatic pathways that drive PL remodelling.

By the late 1990's, methods that allowed the separation and analysis of molecular species of PL had provided details on tissue specific composition, primarily in macrophages and liver ([11–17]). Studies had uncovered high proportions of ether-containing PLs in white blood cells including macrophages [18], and the potent signalling mediator, platelet activating factor, an unusual ether PC, generated via the Lands cycle was known [11, 18, 19]. It had been recognised that the primary route of arachidonate (20 : 4) introduction into PL was via the Lands cycle and that most PC and PE in liver are diacyl species [18], which contain large amounts of this FA at the Sn2 position [11, 14, 16]. The molecular compositions of phosphatidylinositol (PI) and phosphatidylserine (PS), also containing primarily stearate (18 : 0) and 20 : 4 were known [11]. The elucidation of PL biosynthetic pathways proceeded in parallel to the study of the Lands Pathway, and it became clear that PL recycling could occur via at least four sets of enzymatic transformations. These included (i) Acyl-CoA : lysoPL acyltransferases and phospholipaseA2 (PLA2), (ii) CoA-dependent transacylation reactions, (iii) lysophospholipase/transacylase and (iv) CoA-independent mechanisms, which use FA esterified at the sn2 position of a diacylPL as substrate (Figure 2). For a comprehensive review of the state of the art at that time see Yamashita [11]. These different processes are exquisitely controlled in a tissue and cell dependent manner. For example, the Acyl-CoA : lysoPL acyltransferase pathway is widely distributed and most often located on microsomal and plasma membranes. Here, a FA-CoA is esterified into either Sn1 or Sn2 of a lysoPL. Specificity of this reaction for saturated FA at Sn1 and unsaturated FA at Sn2 was shown [3]. Along with Lands, research by Okuyama, Kano and Ohno in Japan elucidated substrate specificity *in vitro* and *in vivo* for PC [20–22]. Complimentary studies on PI and PS were also undertaken [23–25]. Aside from mapping out composition, seminal studies demonstrated that phospholipaseA2 activities play important roles in regulating release of 20 : 4 in cells such as platelets and macrophages in response to calcium ionophore. The second pathway, CoA-dependent transacylation, involves transfer of a FA from a PL, into a lysoPL, via a CoA intermediate. This process has been proposed to not involve free FA formation from PL but a direct formation of FA-CoA from PL metabolism. Many studies on this pathway were undertaken from 1979–1995, and are referenced in [11]. The third pathway involves lysophospholipases which have been shown to catalyse transacylation between two lysoPC, in which one is transferred a FA, to become a PC moiety. Some reports have shown that PLA2 can have acyltransferase and transacylase activities, and maybe involved in PL remodelling [26]. Last, CoA-independent transacylation was shown in cells that contain high levels of ether PL. Here, FA are transacylated from diacyl PL to several different lysoPL in the absence of any cofactors. Generally, this process favours longer chain PUFA [27–31].

While great strides had been made in elucidation of biochemical processes regulating PL recycling, at that time, the identities of the enzymes themselves was limited. Protein purification by detergent solubilisation, chromatography and SDS–polyacrylamide gel electrophoresis had been applied to the analysis of PC and PI acyltransferases in bovine heart [11, 32, 33], but the identities of other proteins were still largely unknown. The molecular biology revolution was only beginning, and researchers looked forward to the application of cDNA cloning approaches which would help identify the key genes and their products involved in PL remodelling.

## Elucidating the enzymes involved using molecular biology approaches

During the 2000's, following the sequencing of genomes and once candidate genes were identified, many products of cDNAs were tested for enzymatic function relating to phospholipid remodelling. Enzymes that were called lysophospholipid acyltransferases (LPLATs) were suggested to be organised into two main families of proteins, then termed lysophosphatidic acid acyltransferases (LPAATs) and membrane bound O-acyltransferases (MBOATs) [34–46]. For readers interested in a comprehensive recent review of the area including full descriptions of studies that characterised cloned proteins *in vitro* please see [47]. However, the naming conventions that were used to refer to LPLATs over the decades created significant confusion in the field. Multiple names have been used for the same enzyme (e.g. based on their substrate preferences), or in other cases, the same name was used for different gene products. To address this, a new nomenclature was recently proposed, based on designating all the enzymes LPLATx, where x denotes the order of discovery, and no information on polar headgroup or substrate is provided. The proteins are then assigned into either MBOAT or AGPAT families [47]. Notably, most enzymes previously termed LPAAT are members of the AGPAT family. In this new system, LPLATs comprise 11 of the 15 members of the AGPAT family and 4 of the 11 members of the MBOAT family, with additional members of both families encoding proteins that are involved in other lipid metabolic processes, such as protein palmitoylation or glycerolipid biosynthesis [47].

### AGPAT family

AGPAT designated LPLATx members are named LPLAT1-10 and 9b, based on the new nomenclature. They have four conserved motifs which based on AlphaFold predictions are believed to define their enzyme active site and recognition of substrate. LPLAT8 and 9 are considered monotopic membrane proteins, with LPLAT8–10 predicted to be anchored via N-terminal helices. These LPLATs are widely expressed in mammalian tissues, and are mainly localised to the ER membrane, although some are also found on Golgi, mitochondrial, nuclear membrane or lipid droplets [47]. As examples of AGPAT family members, the first cloned enzyme shown to generate PC-containing 20:4 was LPLAT9 (LPCAT2) while a reduction in PC-20:4 was shown to occur in LPLAT12 (LPCAT3/MBOAT5)-deficient mice [48–50]. The cloning of LPLAT12 was first reported in 2008 in two closely published studies [46, 51]. See later for a description of the role of LPLAT8 and 9 in inflammatory processes through their role in generation of platelet activating factor (PAF).

### MBOATs

MBOATs with LPLATx activity comprise LPLATs11–14, and they share four conserved motifs, that are distinct to those found in AGPAT family members. These LPLATs have several transmembrane domains, and a conserved histidine suggested to be the active site residue [52]. There are three overall categories of MBOAT based on their enzymatic function, with only one catalysing PL remodelling (LPLATx group). Other MBOATs include ACAT(or SOAT)1,2 and DGAT1 which are involved in neutral lipid biosynthesis, and a group (HHAT, Porcupine, MBOAT4 HHATL) which acylate proteins/peptides. The biochemical reactions are summarised in [53]. Relating to the characterisation of these proteins, cloned enzymes were originally tested for activity using a radiochemical approach, with a single FA-CoA and a single lysoPL [54]. Due to this, previously, the proteins were named by the PL headgroup substrate that is acylated, e.g. LPEAT acylates lysoPE, while LPCAT acylates lysoPC. With the advent of mass spectrometry benchtop instruments, that could be applied to lipidomics, newer assays that allowed choices of multiple FA-CoA esters and lysoPLs could be compared with determine individual substrate specificities [41, 55]. This revealed that single enzymes can often acylate more than one PL headgroup. Thus to reduce confusion, the LPLATx members of the MBOAT family are now all simplified to LPLATs [47]. The lipidomics approach was extended to longer chain PUFA-CoA in a study from Murphy in 2005, which revealed the presence of many LPLAT isoforms in RAW macrophage microsomes [34]. A review on LPLATs and their generation of membrane diversity from Shindou and Shimizu is published here [56].

Beyond lysoPL acyltransferases, when PL recycling involves a fatty acyl-CoA, other enzymes are involved. These include phospholipases (PLA1, PLA2), and also Co-A ligases, which themselves also comprise large families of structurally related proteins showing a high level of tissue specificity and differential regulation of gene expression. First, by hydrolysing FA from a PL, phospholipases provide the substrate for acyl-CoA formation. Human PLA2 enzymes which cleave FA at Sn2 are termed Groups I-VII, X and XII, with several having numerous members. These groups comprise many structurally related proteins expressed by diverse cell types, with some showing calcium sensitivity, while others are secretory forms. Many are actively involved in providing FA substrate for eicosanoid and prostaglandin biosynthesis, as well as PL recycling. PLA1 on the other hand, cleaves FA at Sn1. The best known, PLA1A removes FA from PS, generating lysoPS and is secreted from human platelets [57]. A full description of these is beyond the scope of this review.

The generation of fatty acyl-CoAs is catalysed by a large family of enzymes, called acyl-CoA synthetases (ACS). There are ∼26 mammalian ACS isoforms, which differ in their preference for FA substrates based on chain length [58, 59]. The most relevant for Lands pathway, are the 11 which metabolise FA of between 12–24 carbons, termed long chain acyl-CoA synthetases (ACSL) and very long chain acyl-CoA synthetases (ACSVL), which are also called fatty acid transport proteins (FATP). Five ACSLs metabolise FA between 12–20 carbons, called ACSL1,3,4,5,6. These are distinguished by their tissue and subcellular distribution, FA chain length preferences [60]. ACSVLs comprise six members, called ACSVL 1–6, and they can acylate FAs of up to 24 carbons in chain length [61]. These enzymes not only support acyl-CoA dependent Lands Pathway PL recycling, but also feed in CoA substrates into a huge range of activities, including biosynthesis of glycerides, sterol esters, retinal esters biosynthesis, as well as FA β-oxidation, elongation and desaturation, and protein acylation. See here for two comprehensive reviews of these enzymes, their biochemistry and biology [60, 62].

## The participation of Lands pathway in development, immunity and inflammation

Although the Lands pathway was discovered decades ago, for a long time it was mainly studied from the point of view of its underpinning biochemical mechanisms. More recent research is shedding light on key roles of this fundamental cycle in human health and disease, particularly in development and innate immunity. Indeed, disruption of metabolism of lysoPL via Lands and closely related pathways is associated with several diseases, such as atherosclerosis, vascular dementia and Alzheimer's disease [63–67].

### Development

Studies using knockout mice have revealed underpinning roles of MBOAT family members in biology. The lack of LPLAT11 (MBOAT7) in mice, which is specific for lysoPI, and prefers 20:4 as FA substrate, leads to disordered cortical lamination and delayed neuron migration, and novel loss of function mutations were associated with intellectual disability [68, 69]. Mice lacking MBOATs 1,2 and 5 (LPLAT14,13 and 12 respectively), are available from Jackson Laboratories and are reported to have phenotypic alterations that include: behaviour/neurological (LPLAT14), behavioural, growth, hematopoietic, immune and nervous alterations (LPLAT13) and digestive, growth, metabolic, liver and ageing (LPLAT12) (http://www.informatics.jax.org/). In relation to liver disease, several studies have seen that LPLAT11 (MBOAT7) mutations are associated with development and severity of non-alcoholic fatty liver disease (NAFLD) [70–72], while mice selectively lacking liver LPLAT12 (MBOAT5) show many hepatic alterations including development of steatosis on a chow diet [50] (http://www.informatics.jax.org/diseasePortal/genoCluster/view/57 246). Along with this, mice globally lacking MBOAT5 die soon after birth and also show significant alterations in liver glyceride levels [49, 50]. To date, the detailed mechanistic reasons for these phenotypes are not fully characterised. In a final example, studies using mice show that LPLAT8 is a major source of lung surfactant PC, and that its genetic deficiency leads to respiratory dysfunction [73, 74].

### Innate immunity

A relatively recent finding has been the involvement of Lands Pathway enzymes in the generation of oxygenated PL species, following agonist activation of circulating blood cells such as platelets and neutrophils [75]. As one example, thrombin activation of platelets causes rapid activation of phospholipase A2, oxygenation of PUFA such as 20 : 4 to form various products, followed by their acyl-CoA-dependent esterification into lysoPL to form relatively abundant oxygenated PL [76, 77]. In platelets, the most predominant of these products, termed enzymatically-oxidised PL (eoxPL) to distinguish them from well-known oxidised PL (oxPL), are PE or PC molecular species that contain 12S-hydroxyeicosatetraenoic acid (HETE) which has been generated via 12-lipoxygenase (LOX). These lipids are formed rapidly on cell activation, in the same timescale as formation of free oxylipins, suggesting that the enzyme activities are tightly coupled. Oxylipin-containing PL can also be generated by macrophages, although in the case of 15-LOX in humans or 12/15-LOX in mice, this generally involves direct PL oxidation rather than a Lands pathway process [76, 78, 79]. However, supplementation of RAW macrophages with exogenous free HETE, or HETE-esterified to cholesterol can result in a slower rate of HETE-PL formation, via fatty acyl-CoA intermediates, over a period of a few hours [80, 81]. A recent study showed that esterification via Lands Pathway can mediate removal of large amounts of 12-HETE by RAW macrophages *in vitro* [81].

eoxPL formation appears to be a central part of innate immunity, since the cells that generate them are all essential to our immediate response to acute injury and trauma. Platelets, neutrophils, monocytes and eosinophils all form eoxPL acutely following agonist activation, and recent studies have shown that these lipids may play key roles in the innate immune response itself, reviewed in [75]. EoxPL, in contrast with oxylipins, remain cell associated following their synthesis. This results in sufficient amounts present in cell membranes to have an impact on protein association. In this regard, a role for eoxPL in coagulation has been demonstrated *in vitro* and *in vivo*, where the calcium dependent association of factors with PS is significantly enhanced by the presence of HETE-PLs in the membrane itself [82, 83]. Here, it is proposed that the electronegative -OH group on the eoxPL push the membrane apart, facilitating interactions of calcium ions with the PS headgroup, and enhancing the ability of coagulation factors to associate with the plasma membrane of platelets or eosinophils. Mice lacking either 12-LOX or 12/15-LOX both show a bleeding disorder and generate smaller thrombi on challenge, as well as being resistant to inflammatory vascular disease such as atherosclerosis and abdominal aortic aneurysm [84–88]. The most abundant eoxPL are HETE-containing forms, followed by those containing monohydroxy-FA from other PUFA [89]. This is most likely due to the fact that these are quantitatively the most abundant oxylipins generated during blood clotting. However, using LC–MS/MS, ∼100 individual species could be detected on platelet activation, with many containing multiple oxygenations, but only so far being partially structurally characterised [89]. A major challenge in studying these lipids is the lack of synthetic standards, with only a small number being commercially available, as well as the significant structural complexity of products formed. The specific MBOATs/ACSLs specifically involved in formation of eoxPL in immune cells are so far uncharacterised. A study by Klett et al. demonstrated that several ACSLs can recognise oxygenated PUFA *in vitro*, using recombinant enzymes [90].

### Formation and metabolism of lysoPL

Aside from their role as substrates for PL recycling, lysoPL are bioactive lipids that signal through interacting with G protein coupled receptors [91–93]. They also appear to alter membrane properties and interact directly with proteins [66, 94–96]. They have recognised roles in inflammatory disease, cancer and many other conditions and thus, regulation of their levels via enzymatic metabolism is critical. LysoPL directly promote Ras-mediated activation of MAPK, cell-cycle progression and differentiation and their plasma concentrations are high, ∼200 µM. The Lands Pathway is a major source of lysoPL, and its removal, although in many cells, following formation of lysoPL, they are mainly removed by lysophospholipases. In this regard, a recent study by Marnett characterised the substrate specificity of lysophospholipases in murine neuroblastoma [97].

### LPLAT8 and 9 (LPCAT1 and 2): roles in inflammation and cancer

PAF is an unusual phosphatidylcholine species which is characterised by a plasmalogen bond at *Sn1* and an acetate bound at *Sn2*. Several studies have shown an essential role for two LPLAT isoforms in regulating its formation, namely LPLAT8 and 9, formerly known as LPCAT1 and 2 respectively. LPLAT8 was proposed to be involved in formation of noninflammatory PAF [98], while LPLAT9 is known to acylate acetic acid (C2:0) into lyso-PAF, to generate PAF [48, 99], involved in inflammation. While LPLAT8 is considered constitutively expressed, LPLAT9 is activated by phosphorylation in response to stimuli such as lipopolysaccharide, which is also capable of inducing it in macrophages. LPLAT9 has a proposed role in regulating neuropathic pain via PAF production [100] and a proposed role in IgE overproduction in allergy [101]. Both LPLAT8 and 9 have several reported roles in cancer progression and resistance to chemotherapy [102–106]. For more detail, please see review [47].

## Conclusion

The Lands Pathway has been known about since the end of the 1950's, when it was first noted by Bill Lands that a distinct metabolic pathway for PL recycling existed. This is one of the key processes by which membrane lipid composition is controlled across tissues and cells. Since then, our knowledge of the function and importance of the enzymes and products of this pathway has grown significantly and study of the role of lysoPL themselves in health and disease, and novel oxygenated PLs generated by this cycle is a major focus of ongoing research. With the advent of new generation LC/MS/MS methods, revisiting Lands pathway enzyme specificities in complex tissues in health and disease would be timely, since older studies mainly characterised their biochemistry using cloned enzymes. Currently in most studies, only existing levels of lipids are measured, with no analysis of their rapid dynamics of formation and degradation. This is a major disadvantage of current methods, and one example of an approach that addresses this gap is shown here in the case of a time resolved analysis of hepatocyte glyceride metabolism [107]. One topical area where their contribution maybe understudied and is of likely importance is in the replication of enveloped viruses, such as SARS, MERS, influenza, HIV and many others. Another area that is ripe for development is flux analysis. It is well known that viral infection leads to the exploitation of host cell lipids and their synthetic machinery, to support the PL biosynthesis needed for virus assembly and egress (referenced in [108]). The exact role of Lands Pathway in these events deserves study since it may represent a target for anti-viral strategies.

## Perspectives

- The Lands Pathway is a fundamental underpinning biochemical process, first elucidated in the 1950's.
- It is required for membrane biogenesis, and also formation of bioactive lipid mediators, and controlled in a cell and tissue dependent manner by numerous related gene products.
- New appreciation of the Lands Pathway in cell biology and immunology is being realised through lipidomics studies, and future directions will reveal mechanistic insights into the detailed roles of this cycle in human health and disease.

## Open Access Statement

Open access for this article was enabled by the participation of Cardiff University in an all-inclusive Read & Publish pilot with Portland Press and the Biochemical Society under a transformative agreement with JISC.

## Abbreviations

ACS: acyl-CoA synthetases
ACSVL: very long chain acyl-CoA synthetases
FA: fatty acyl
HETE: hydroxyeicosatetraenoic acid
PAF: platelet activating factor
PI: phosphatidylinositol
PL: phospholipids
PLA: phospholipaseA2
PS: phosphatidylserine
